# Does advancement in marker-less pose-estimation mean more quality research? A systematic review

**DOI:** 10.3389/fnbeh.2025.1663089

**Published:** 2025-08-22

**Authors:** Shivam Bhola, Hyun-Bin Kim, Hyeon Su Kim, BonSang Gu, Jun-Il Yoo

**Affiliations:** ^1^Department of Orthopedic Surgery, Inha University Hospitals, Incheon, Republic of Korea; ^2^Department of Biomedical Sciences, College of Medicine, and Program in Biomedical Science & Engineering, Inha University, Incheon, Republic of Korea; ^3^Department of Biomedical Research Institute, Inha University Hospitals, Incheon, Republic of Korea

**Keywords:** marker-less pose estimation, keypoint detection, behavior classification, rodent model, systematic review

## Abstract

Recent breakthroughs in marker-less pose-estimation have driven a significant transformation in computer-vision approaches. Despite the emergence of state-of-the-art keypoint-detection algorithms, the extent to which these tools are employed and the nature of their application in scientific research has yet to be systematically documented. We systematically reviewed the literature to assess how pose-estimation techniques are currently applied in rodent (rat and mouse) models. Our analysis categorized each study by its primary focus: tool-development, method-focused, and study-focused studies. We mapped emerging trends alongside persistent gaps. We conducted a comprehensive search of Crossref, OpenAlex PubMed, and Scopus for articles published on rodent pose-estimation from 2016 through 2025, retrieving 16,412 entries. Utilizing an AI-assisted screening tool, we subsequently reviewed the top ∼1,000 titles and abstracts. 67 papers met our criteria: 30 tool-focused reports, 28 method-focused studies, and nine study-focused papers. Publication frequency trend has accelerated in recent years, with more than half of these studies published after 2021. Through a detailed review of the selected studies, we charted emerging trends and key patterns, from the emergence of new keypoint-detection methods to their integration into behavioral experiments and adoption in various disease contexts. Despite significant progress in marker-less pose-estimation technologies, their widespread application remains limited. Many laboratories still rely on traditional behavioral assays, under-using advanced tools. Establishing standardized protocols is the key step to bridge this gap, which will ultimately realize the full potential of marker-less pose-estimation and even greater insight into preclinical behavioral science.

## 1 Introduction

The fate preclinical behavioral science relies heavily on early *in vivo* experimentations. Detailed quantification of rodent behavior is essential for understanding disease progression, and treatment efficacy (Lauer et al., 2022). Traditionally, researchers have relied on simple, manual assays such as timing a mouse’s pause before exploration or counting how often it crosses grid lines that require human observers to note every action. Besides being tedious and prone to bias, these manual approaches usually miss subtle micro-behaviors such as tiny head lifts, brief standing events, or slight changes in stride that can contain critical clues about early pathological signs (Miller et al., 2011; Desland et al., 2014). Now the question is about the way to detect those subtle micro-behaviors.

Over the last ten years, deep-learning–powered, marker-less pose-estimation has transformed behavioral analysis by detecting key anatomical points like the snout, paws, and tail from video footage without any physical markers. The DeepLabCut (DLC) software (2018) achieved human-level accuracy in tracking fast-moving rodents at the pixel scale trained using 50–200 manually labeled frames (Mathis et al., 2018). Following this innovation, a broad list of tools has emerged, including, AlphaTracker (Chen et al., 2023), DeepLabStream (Schweihoff et al., 2021), Keypoint-MoSeq (Weinreb et al., 2024), and Social LEAP Estimates Animal Poses [SLEAP (Pereira et al., 2022)].

Although these pose-estimation tools have advanced rapidly, their adoption in standard rodent research workflows remains sporadic and largely undocumented. Many labs still depend on hand-scored tests, missing out on the detailed, high-resolution data that automated pose-estimation algorithms can offer. Key contributing factors include: (1) operational costs such as retraining personnel, reconfiguring equipment, and provisioning computational resources require significant effort (Hagelskjær et al., 2018); (2) technical complexity such as diverse pose-estimation packages demand different installations and parameter settings, non-expert users can become easily overwhelmed (Dubey and Dixit, 2023); (3) a lack of standards leaving users without clear guidelines for evaluation tools (Reed et al., 1999); and (4) video-data challenges, the burden of archiving large datasets and ensuring analyses can be reliably repeated (Nassauer and Legewie, 2019).

To map the current state of adoption, we systematically reviewed studies published in last 10 years by searching four databases Crossref, OpenAlex, PubMed, and Scopus for reports of marker-less, keypoint-based tracking in rat and mouse models. Using an AI-assisted screening process, we narrowed approximately 16,412 initial hits to 63 relevant articles and four Tool-focused papers manual included. Each was classified into one of three categories: tool-focused, method-focused, or study-focused study and we mapped key trends and remaining challenges.

Our specific aims were to (1) quantify publication trends and the prevalence of different software platforms; (2) classify research by its primary aim and experimental setting [such as locomotion assays (Sturman et al., 2020), social behavior tests (Sterley et al., 2024), and anxiety paradigms (Sharma et al., 2025)]; (3) map the behavioral assays and disease contexts [such as Parkinson’s (Andreoli et al., 2021), Alzheimer’s (Miller et al., 2024), and pain models (Li et al., 2024)] that have used pose tracking; and (4) highlight gaps and opportunities. Through this in-depth analysis, we aim to assess whether the accuracy, flexibility, and ease of use of current pose-estimation tools have been broadly adopted in *in vivo* research, as well as the gaps between innovative technologies and standard preclinical behavioral science.

## 2 Materials and method

Following PRISMA (Preferred Reporting Items for Systematic Reviews and Meta-Analyses) guidelines, we systematically reviewed only publications focused on rodent marker-less pose-estimation techniques. Below, we detail the protocol we followed for the search strategy, eligibility criteria, study selection, data extraction, risk of bias assessment, and data synthesis

### 2.1 Search strategy

We performed a comprehensive literature search to identify studies involving marker-less pose-estimation in rodent (rat or mouse) models. The search covered publications from January 2016 up to March 2025. We constructed search queries using combinations of keywords related to pose-estimation (such as “pose-estimation,” “posture tracking,” “keypoint detection,” “behavioral tracking”) and rodents (such as “rodent,” “rat,” “mouse,” “mice”), as well as specific tool names known in the field (such as “DeepLabCut,” “LEAP”, “SLEAP,” etc.). These searches were run across multiple databases and search engines, including Crossref, OpenAlex PubMed, and Scopus. To facilitate broad retrieval, we utilized the Publish or Perish software to query those databases with standardized search strings (Harzing, 2007). The search was limited to the English language literature. The final search was completed on 18 March 2025. All references retrieved were imported into a reference manager, and duplicate entries were removed prior to screening. The process was completely automated without any human intervention.

### 2.2 Eligibility criteria

Studies identified from the search were evaluated against predetermined inclusion and exclusion criteria:

Inclusion criteria: (1) Studies must involve marker-less pose-estimation (keypoint-based tracking of body parts) performed on rodents (rats or mice). (2) The pose-estimation should be applied to data from an experimental or observational study (i.e., the researchers collected or used rodent behavioral data in which pose tracking was implemented). (3) The pose tracking must involve more than one key point (to exclude cases like single-point tracking of an animal’s centroid). (4) Articles must present original data rather than hypothetical concepts.

Exclusion criteria: (1) Review papers, editorials, or meta-analyses were excluded (we only included primary research studies). (2) Studies focusing on non-rodent species or on humans were excluded, even if they discussed pose-estimation, to keep the scope specific to rodent models. (3) Studies that did not actually perform keypoint detection – for example, those that only discuss pose-estimation conceptually or use other tracking methods (like bounding box or mask) without implementing a keypoint algorithm – were excluded. (4) If a study solely used marker-based motion capture or sensor-based tracking (and not marker-less pose-estimation), it was excluded.

### 2.3 Study selection

**FIGURE 1:**
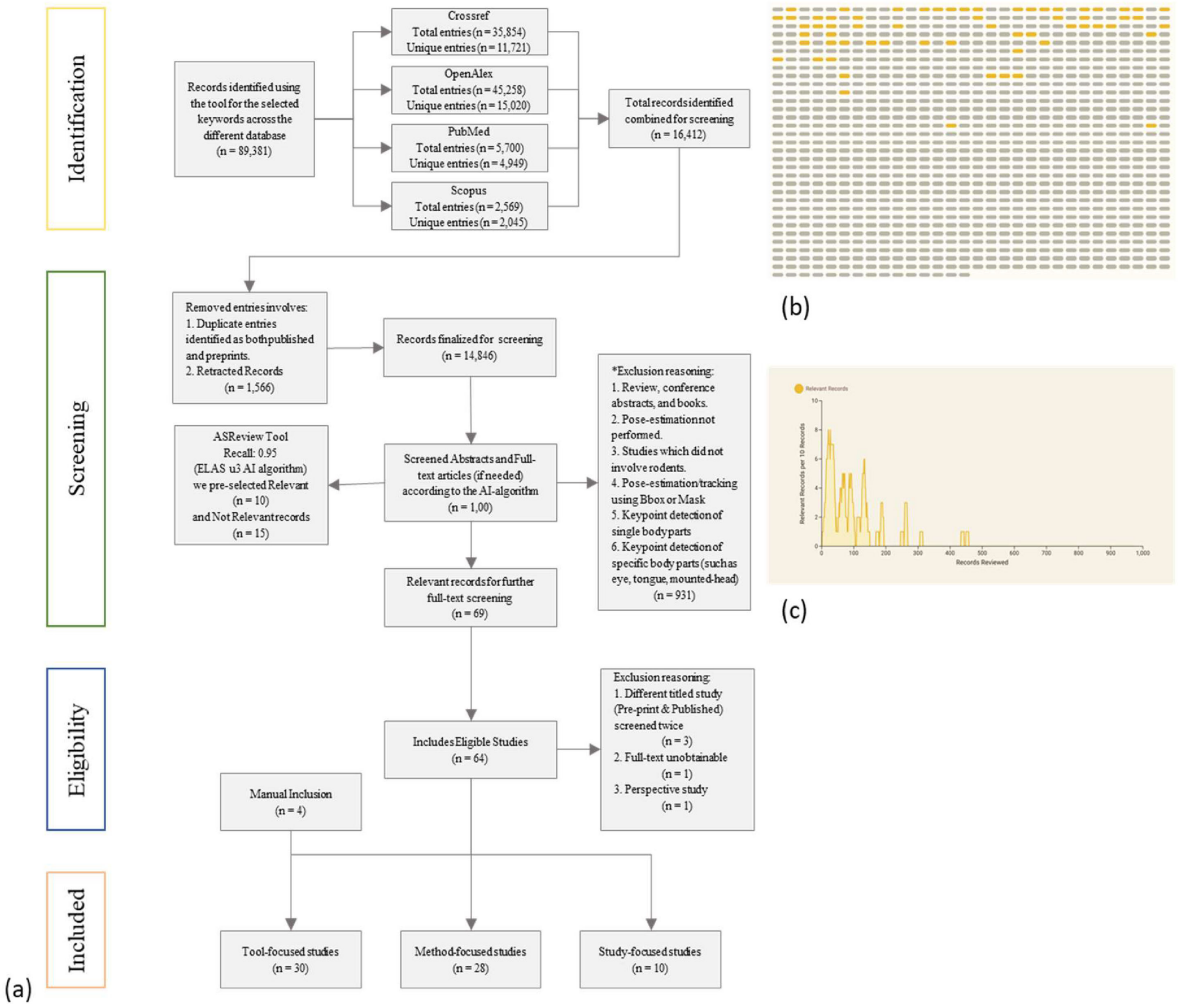
PRISMA flow chart **(a)** *n* = 16,412 studies were retrieved; out of which top *n* = 1,000 studies were screened using ASReview Tool. Full-text of Relevant records *n* = 69 were manually screened for the selection of the 30 Tool-focused (*n* = 4 Manual Inclusion), 28 Method-focused, and nine Study-focused included studies; *as the screening process is AI-assisted the quantitative details of excluded studies are unavailable. The ASReview screening result is represented as **(b)** the relevant studies found in chronological order and **(c)** rate of relevant record discovery.

The study selection process summarized in the flow diagram (Figure 1). First, titles and abstracts of all retrieved records were screened to exclude those unrelated to our focus.

To assist with screening and ensure, we employed the machine learning tool ASReview (de Bruin et al., n.d.), which prioritized records based on the likelihood of relevance. Using this tool, we iteratively screened approximately 1,000 articles by title, abstract, and full-text (if needed). This process allowed us to quickly identify candidate studies; any reference that the AI model flagged as relevant was given careful consideration, to maintain sensitivity (recall: 0.95). This process was primarily automated using advanced AI method with minimal human intervention. After initial screening, we obtained the full texts of studies that were potentially eligible. Authors then reviewed each full text in detail to determine final inclusion.

For the analysis, studies included in the qualitative synthesis; were categorized into one of three groups based on its primary focus: (1) Tool-focused papers: Studies primarily centered on the development or validation of a pose-estimation tool or algorithm. These typically introduced new software, frameworks, or technical improvements for tracking and analyzing rodent posture/behavior. (2) Method-focused papers: Studies that applied pose-estimation to propose a specific experimental method or paradigm. These papers often aimed to demonstrate how pose tracking can enhance a particular behavioral test or an experimental setup using pose-estimation. (3) Study-focused papers: Studies that used pose-estimation within the context of addressing a biological or disease-related research interest.

All of the included studies outlined a solution leveraging marker-less pose-estimation. Despite similarities in their methodologies and overlapping theme, we tried to categorized them based on the best-fitting approach.

### 2.4 Data extraction

Data of interests were manually pulled by authors from each included article using a preformatted form in an Excel workbook. For the tool-development paper, recorded fields included tool name, primary function or novelty, publication date, computational, code availability, and reported performance metrics across. For Method and Study papers, we extracted information on the experimental design and context: the rodent species/strain and any disease model or condition, the behavioral tests or tasks conducted, the pose-estimation software or method used, the number of key points tracked, the performance of tools, and the main outcomes or findings related to pose. The compiled data are organized into category-specific tables (Supplementary Tables 1–3).

### 2.5 Risk of bias assessment

We evaluated each study’s risk of bias to maintain the trustworthiness of the findings. Given that the selected studies involved rodent experiments and computation analyses, in accordance with the ROBINS-I-V2 framework (for non-randomized studies), the risk of bias assessment was performed. Due to the nature of the current study, certain bias criteria were not applicable, but we still noted them according to the guidelines. The seven domains of bias include: Bias due to confounding; Bias in selection of participants into the study; Bias in classification of interventions; Bias due to deviations from intended interventions; Bias due to missing data; Bias in measurement of outcomes; and Bias in selection of the reported result. Each study was evaluated across multiple domains and assigned a risk level of “low risk,” “high risk,” or “unclear risk,” of bias. The risk of bias findings was compiled into summary tables and visualized via the Robvis tool (McGuinness and Higgins, 2021). These figures display the proportion of low/moderate/serious/critical risk in each domain and provide an individual risk profile for each paper.

### 2.6 Data synthesis

No meta-analysis was performed considering the study designs. Or rather, due to lack of the standardized benchmark across the marker-less pose-estimation field. Instead, we created a qualitative, descriptive review of the findings, structuring our results to align with the review objectives and presenting summary tables and figures to illustrate the main idea. Specifically, we synthesized the findings through (1) narrative summaries for each category (Tool, Method, Study), detailing their shared themes, technological advancements, and primary results. (2) We generated a year-by-year publication chart for tool-development studies and for method-application studies to track adoption patterns over time. (3) We classified pose-estimation tools according to their primary functions to illustrate the field’s functional diversity. (4) We analyzed the technical aspects across tools (such as network backbones) and alongside experimental variables in method and study papers (identifying the most frequently used behavioral assays and disease models in pose-estimation research). (5) Where applicable, we compared performance metrics (such as accuracy and processing speed) across tools where data permitted. Observed trends and gaps found are highlighted across the study.

## 3 Results

### 3.1 Study selection

The comprehensive search across the selected sources yielded a large number of references. The PRISMA flow diagram in Figure 1a traces the study selection pathway from the initial database search through each subsequent screening stage. After removing duplicates, a total of 16,412 unique records were screened using ASReview (de Bruin et al., n.d.). Figure 1b represents the chronological record and Figure 1c the rate of relevant studies discovered using ASReview tool, which is the reason to screen the top 1,000 suggested records by title and abstract and full-text if needed. As shown, the recall of relevant studies falls well within this range, indicating a low likelihood of missing key studies. Of those, identifying 69 that were found to be relevant to the current study. The relevant studies were then advanced to full-text screening find 63 that met our inclusion criteria and were analyzed qualitatively. We classified these into three groups: 26 Tool-focused papers, 28 Method-focused papers, and nine Study-focused papers. In addition, we have included four Tool-focused papers manually, which were overlooked during screening.

### 3.2 Characteristics of the included studies

The key characteristics of all 67 included studies were summarized across three tables corresponding to Tool, Method, and Study. In the following sections, we detail each table’s contents and discuss the key findings. Notably, each study confirms the added impact of pose-estimation in research on disease models.

Tool-focused studies (*N* = 30): Table 1 provides an overview of the studies focused on keypoint or behavior detection employing marker-less pose-estimation techniques. These studies predominantly introduce new pose-estimation frameworks or significant extensions to existing ones. The entries in list each tool’s given name, the publication year, and running title is mentioned.

**TABLE 1:** List of selected studies in the category tool-focused (*n* = 30).

Name of tool	Year	Title
DeepLabCut (Mathis et al., 2018)	2018	DeepLabCut: marker-less pose-estimation of user-defined body parts with deep learning
LEAP (Pereira et al., 2019)	2018	Fast animal pose-estimation using deep neural networks
DeepPoseKit (Graving et al., 2019)	2019	DeepPoseKit, a software toolkit for fast and robust animal pose estimation using deep learning
SimBa (Nilsson et al., 2020)	2020	Simple Behavioral Analysis (SimBA): an open source toolkit for computer classification of complex social behaviors in experimental animals
Anipose (Karashchuk et al., 2021)	2021	Anipose: A toolkit for robust markerless 3D pose estimation
CAPTURE (Marshall et al., 2021)	2021	Continuous Whole-Body 3D Kinematic Recordings across the Rodent Behavioral Repertoire
DANNCE (Dunn et al., 2021)	2021	Geometric deep learning enables 3D kinematic profiling across species and environments
DeepLabStream (Schweihoff et al., 2021)	2021	DeepLabStream enables closed-loop behavioral experiments using deep learning-based marker-less, real-time posture detection
MARS (Segalin et al., 2021)	2021	The Mouse Action Recognition System (MARS) software pipeline for automated analysis of social behaviors in mice
MouseVenue3D (Han et al., 2022)	2021	MouseVenue3D: A Marker-less Three-Dimension Behavioral Tracking System for Matching Two-Photon Brain Imaging in Free-Moving Mice
OptiFlex (Liu et al., 2021)	2021	OptiFlex: Multi-Frame Animal Pose-estimation Combining Deep Learning With Optical Flow
BehaviorDEPOT (Gabriel et al., 2022)	2022	BehaviorDEPOT is a simple, flexible tool for automated behavioral detection based on marker-less pose tracking
DeepLabCut (Lauer et al., 2022)	2022	Multi-animal pose-estimation, identification and tracking with DeepLabCut
SLEAP (Pereira et al., 2022)	2022	SLEAP: A deep learning system for multi-animal pose tracking
AlphaTracker (Chen et al., 2023)	2023	AlphaTracker: a multi-animal tracking and behavioral analysis tool
DeepOF (Bordes et al., 2023)	2023	Automatically annotated motion tracking identifies a distinct social behavioral profile following chronic social defeat stress
ContrastivePose (Zhou et al., 2023)	2023	ContrastivePose: A contrastive learning approach for self-supervised feature engineering for pose-estimation and behavorial classification of interacting animals
SaLSa (Sakata, 2023)	2023	SaLSa: A Combinatory Approach of Semi-Automatic Labeling and Long Short-Term Memory to Classify Behavioral Syllables
Temporal Semi-supervision method (Li et al., 2023)	2023	Improved 3D Marker-less Mouse Pose-estimation Using Temporal Semi-supervision
A-SOiD (Tillmann et al., 2024)	2024	A-SOiD, an active-learning platform for expert-guided, data-efficient discovery of behavior
ABNet (Chen et al., 2024)	2024	ABNet: AI-Empowered Abnormal Action Recognition Method for Laboratory Mouse Behavior
ARBEL (Barkai et al., 2024)	2024	ARBEL: A Machine Learning Tool with Light-Based Image Analysis for Automatic Classification of 3D Pain Behaviors
FABEL (Catto et al., 2024)	2024	FABEL: Forecasting Animal Behavioral Events with Deep Learning-Based Computer Vision
Keypoint-MoSeq (Weinreb et al., 2024)	2024	Keypoint-MoSeq: parsing behavior by linking point tracking to pose dynamics
Lightning Pose (Biderman et al., 2024)	2024	Lightning Pose: improved animal pose-estimation via semi-supervised learning, Bayesian ensembling, and cloud-native open-source tools
REVEALS (Phadke et al., 2024)	2024	REVEALS: An Open Source Multi Camera GUI For Rodent Behavior Acquisition
STCS (Tang et al., 2024)	2024	Segmentation tracking and clustering system enables accurate multi-animal tracking of social behaviors
STPoseNet (Lv et al., 2024)	2024	STPoseNet: A real-time spatiotemporal network model for robust mouse pose-estimation
SuperAnimal Model (Ye et al., 2024)	2024	SuperAnimal pretrained pose-estimation models for behavioral analysis
Seizure Classification Pipeline (Yu et al., 2025)	2025	Integrating manual preprocessing with automated feature extraction for improved rodent seizure classification

Method-focused studies (*N* = 28): Table 2 summarizes the studies that we categorized as Method papers. These works employed existing pose-estimation techniques to advance or refine a particular experimental method in behavioral research. For each study, we list the name of method, behavioral test that was the focus (such as balance beam walking test, open field test, elevated plus maze, operant conditioning task, etc.), year of publication, and the running title.

**TABLE 2:** List of selected studies in the category method-focused (*n* = 28).

Name of method	Behavior test	Year	Title
DLCAnalyzer (Sturman et al., 2020)	Open-field; Elevated Plus Maze; Forced Swim Test	2020	Deep learning–based behavioral analysis reaches human accuracy and is capable of outperforming commercial solutions
*Not Specified* (Lang et al., 2020)	Unrestricted cage exploration Rodent Beam Walk	2020	Detecting and quantifying ataxia-related motor impairments in rodents using marker-less motion tracking with deep neural networks
Marker-less 2D Kinematic Analysis (Sato et al., 2022)	Treadmill walking	2021	Marker-less analysis of hindlimb kinematics in spinal cord-injured mice through deep learning
PS-VAE (Whiteway et al., 2021)	Head-fixed tasks Open Field Test	2021	Partitioning variability in animal behavioral videos using semi-supervised variational autoencoders
Automated Deep Phenotyping Pipeline (Klibaite et al., 2022)	Open Field Test	2022	Deep phenotyping reveals movement phenotypes in mouse neurodevelopmental models
VAME (Luxem et al., 2022)	Open Field Test	2022	Identifying behavioral structure from deep variational embeddings of animal motion
*Not Specified* (O’Neill et al., 2022)	Single Pellet Retrieval Task	2022	Marker-less tracking enables distinction between strategic compensation and functional recovery after spinal cord injury
*Not Specified* (Sheppard et al., 2022)	Open Field Test	2022	Stride-level analysis of mouse open-field behavior using deep-learning-based pose-estimation
AMBER (Lapp et al., 2023)	Maternal behavior observation	2023	Automated maternal behavior during early life in rodents (AMBER) pipeline
Posture Analysis Workflow (Wan et al., 2023)	Rodent Beam Walk	2023	An integrated workflow for 2D and 3D posture analysis during vestibular system testing in mice
SEB3R (Chelini et al., 2023)	Open Field Test	2023	Automated Segmentation of the Mouse Body Language to Study Stimulus-Evoked Emotional Behaviors
Two-bottle choice assay (Kim et al., 2023)	Two-bottle choice licking assay in fasted vs. sated mice	2023	Evaluation of mouse behavioral responses to nutritive versus non-nutritive sugar using a deep learning-based 3D real-time pose-estimation system
*Not Specified* (Bogachev et al., 2023)	Open Field Test	2023	Video-based marker-free tracking and multi-scale analysis of mouse locomotor activity and behavioral aspects in an open field arena: A perspective approach to the quantification of complex gait disturbances associated with Alzheimer’s disease
*Not Specified* (Li et al., 2022)	Overground runway gait Treadmill locomotion	2023	A Multimode Marker-less Gait Motion Analysis System Based on Lightweight Pose-estimation Networks
Air-Stepping (Mistretta et al., 2024)	Air Stepping in neonatal mouse	2024	Air-stepping in the neonatal mouse: a powerful tool for analyzing early stages of rhythmic limb movement development
Arthritis Pain Assessment System (Li et al., 2024)	Automated Arthritis Pain Assessment	2024	Combining dual-view fusion pose-estimation and multi-type motion feature extraction to assess arthritis pain in mice
BAS (Piotrowski et al., 2024)	Rodent Beam Walk	2024	Phenotypic analysis of ataxia in spinocerebellar ataxia type 6 mice using DeepLabCut
KineWheelSystem (Albrecht et al., 2024)	Paw placement tracking on the KineWheel	2024	KineWheel–DeepLabCut Automated Paw Annotation Using Alternating Stroboscopic UV and White Light Illumination
MoSoMoTr (Sterley et al., 2024)	Social interaction assays	2024	Marker-less Mouse Tracking for Social Experiments
SBeA (Han et al., 2024)	Free–social behavior	2024	Multi-animal 3D social pose-estimation, identification and behavior embedding with a few-shot learning framework
String Pulling Analysis Pipeline (Sandhu et al., 2024)	Standing string-pulling for reward	2024	Information-theory analysis of mouse string-pulling agrees with Fitts’s Law: Increasing task difficulty engages multiple sensorimotor modalities in a dual oscillator behavior
*Not Specified* (Zahran et al., 2024)	Three Chamber Sociability and Social Novelty Test	2024	Deep learning-based scoring method of the three-chamber social behavior test in a mouse model of alcohol intoxication. A comparative analysis of DeepLabCut, commercial automatic tracking and manual scoring
*Not Specified* (Bidgood et al., 2024)	Rodent Beam Walk	2024	Automated procedure to detect subtle motor alterations in the balance beam test in a mouse model of early Parkinson’s disease
ArguelloALab (Sanabria et al., 2025)	Operant Self-Administration (Cocaine IVSA)	2025	Analysis of Operant Self-administration Behaviors with Supervised Machine Learning: Protocol for Video Acquisition and Pose-estimation Analysis Using DeepLabCut and Simple Behavioral Analysis
ForestWalk (Tozzi et al., 2025)	Rodent Beam Walk	2025	Forestwalk: A Machine Learning Workflow Brings New Insights Into Posture and Balance in Rodent Beam Walking
InteBOMB (Zhai et al., 2025)	Open-Field; Elevated Plus Maze; Forced Swim Test	2025	InteBOMB: Integrating generic object tracking and segmentation with pose-estimation for animal behavior analysis
LBWT-AT (Ruiz-Vitte et al., 2025)	Rodent Beam Walk	2025	Ledged Beam Walking Test Automatic Tracker: Artificial intelligence-based functional evaluation in a stroke model
Touchscreen-based rodent Continuous Performance Test (rCPT) (Li et al., 2025)	Touchscreen-based rCPT	2025	Time-on-task–related decrements in performance in the rodent continuous performance test are not caused by physical disengagement from the task

Study-focused studies (*N* = 9): Table 3 provides details on the included studies that we classified as Study papers, meaning their primary aim was to answer a biological or disease-related question, with pose-estimation being a means to that end. Each entry in table describes the disease or model under investigation, the behavioral assays used, year of publication, and the running title. All records included in the current studies performed the significant role in the advancement of the pose-estimation field.

**TABLE 3:** List of selected studies in the category Studies-focused (*n* = 9).

Condition	Behavior test	Year	Title
Parkinsonism (6-OHDA lesion) (Andreoli et al., 2021)	Abnormal involuntary movements rating	2021	Distinct patterns of dyskinetic and dystonic features following D1 or D2 receptor stimulation in a mouse model of parkinsonism
Mechanical pain hypersensitivity (Zhang et al., 2022)	Home-cage exploration	2022	Automated preclinical detection of mechanical pain hypersensitivity and analgesia
Photothrombotic ischemic stroke (Weber et al., 2022)	Gait analysis	2022	Deep learning–based behavioral profiling of rodent stroke recovery
Thermal pain hypersensitivity (Reddy et al., 2023)	Thermal-plate assay	2023	A Deep-Learning Driven Investigation of the Circuit Basis for Reflexive Hypersensitivity to Thermal Pain
Neuropathic pain (Norris et al., 2023)	Tail suspension assay	2023	Spared nerve injury causes motor phenotypes unrelated to pain in mice
Alzheimer’s disease models (Miller et al., 2024)	Open-field exploration	2024	Machine learning reveals prominent spontaneous behavioral changes and treatment efficacy in humanized and transgenic Alzheimer’s disease models
Rett syndrome (Mykins et al., 2024)	Pup retrieval	2024	Multidimensional Analysis of a Social Behavior Identifies Regression and Phenotypic Heterogeneity in a Female Mouse Model for Rett Syndrome
Dopamine depletion model (Yang et al., 2024)	Open-field locomotion	2024	Dopamine lesions alter the striatal encoding of single-limb gait
Buprenorphine exposure (Sharma et al., 2025)	Elevated zero maze	2025	Machine learning and confirmatory factor analysis show that buprenorphine alters motor and anxiety-like behaviors in male, female, and obese C57BL/6J mice

### 3.3 Risk of bias assessment

We evaluated the risk of bias for the included studies, and a summary is presented in Supplementary Figures 1–3. In general, the methodological quality of studies varied, with many studies showings some risk of bias or reporting limitations in one or more domains. For the subset of studies, common issues included the lack of explicit randomization of animals into experimental groups and blinding. For example, several papers did not clearly state whether the experimenters were blinded to treatment or genotype during behavioral assessments, raising the risk of detection bias. On the other hand, most studies clearly defined their objectives and reported results thoroughly, so reporting bias was generally low. Many studies did not report factors such as animal selection, age, housing conditions, sleep–wake cycles; recording sessions plan likely deemed irrelevant for tool performance. However, overlooking these variables introduces confounding and selection bias. Additionally, some behavior classification studies omitted keypoint detection accuracy, raising concerns of reporting bias. Depending on the context, these biases may impact the reliability of datasets or the development of new tools or methods. In the graphical representation, we provide an aggregate view using color-blind safe format: indicate a low risk, a moderate, a high risk, and unclear risk in each domain. Overall, while no study was excluded due to quality, the assessment suggests that around half of the studies had at least one domain with a potential issue, meaning results should be interpreted with that context in mind. Conversely, about half of the studies (especially some tool papers and well-designed experiments) adequately addressed most bias concerns. It also highlights an area for improvement: future studies, particularly those implementing pose-estimation in biological experiments, should consider a robust design practice to strengthen confidence in their findings.

## 4 Quality evaluation of the included studies

Beyond the basic characteristics and bias assessment, we conducted further analyses to evaluate the trends and qualities of the included studies. In the following subsections, we present these findings, which encompass the temporal trends in publications, the evolution of tool features, the contexts in which pose-estimation is applied, and the usage patterns of different pose-estimation software. Each subsection entertains a specific aspect of idea and is represented by a figure or table.

### 4.1 Chronological list of tools

**FIGURE 2:**
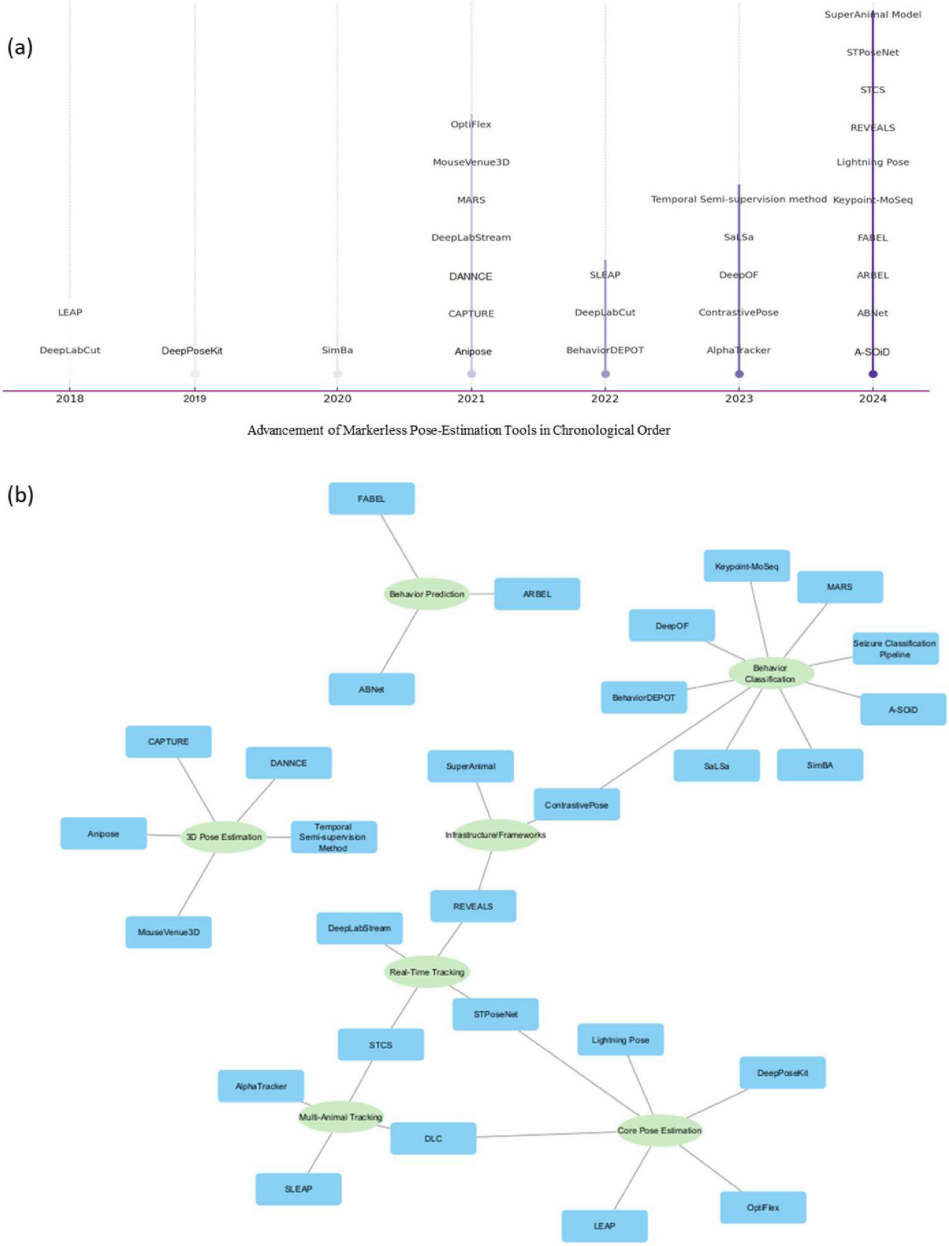
**(a)** Based on the year of publication, the timeline of marker-less rodent pose-estimation based keypoint detection and behavior detection tools. **(b)** The current network represents the primary intended purpose and key capabilities. The main categories (Green node) include: Core Pose-estimation, Multi-Animal Tracking, 3D Pose-estimation, Real-Time Tracking, Behavior Classification, Behavior Prediction, and Infrastructure/Frameworks. The published tools (Blue node) are linked with their intended purpose.

We first examined the timeline of publication related to the rodent pose-estimation tools (Figure 2a). The analysis revealed a clear upward trend over the past several years. From 2018 through 2025, there has been a steady increase in the number of new tools published per year. In the initial period, only a handful of tools were introduced, like DLC (Mathis et al., 2018) and LEAP (Pereira et al., 2019). The pace picked up modestly around 2020–2021 and then surged markedly in 2023 and 2024. The year 2024, in particular, saw the highest influx, with seven distinct new tools reported in that year alone, according to our dataset. This suggests that the field of pose-estimation for rodent analysis is in a phase of rapid innovation.

Several factors likely contributed to this growth: the success and wide adoption of initial tools probably spurred further developments with the increasing computational resources and open-source frameworks have lowered the barrier to creating new specialized tools (Voulodimos et al., 2018). By early 2025, the trend appears to continue, with at least a new tool already published in the first part of 2025, the seizure classification pipeline (Yu et al., 2025). The chronological trend underscores that the technology landscape is evolving quickly, and researchers are actively working on new solutions to extend capabilities. This also means that researchers will get access to an expanding array of tools to choose from with the advancement in the pose-estimation technology.

### 4.2 Primary purpose

We categorized each pose-estimation tool (from the Tool-focused studies) by its primary intended purpose (Figure 2b). This classification helps illustrate the diversity of approaches and end goals among the tools in this field. We identified several broad categories of tool functionality: (1) Core Pose-estimation: Tools whose primary aim is accurate marker-less tracking of animal key points. Examples: DLC (Mathis et al., 2018), LEAP (Pereira et al., 2019), SLEAP (Pereira et al., 2022) (functional successor of LEAP), Lightning Pose (Biderman et al., 2024), STPoseNet (Lv et al., 2024). These focus on improving the accuracy, robustness, or efficiency of pose detection. (2) Multi-Animal Tracking: Tools designed to track multiple animals simultaneously and possibly maintain individual identities. Examples: AlphaTracker (Chen et al., 2023), MARS (Segalin et al., 2021), SLEAP (Pereira et al., 2022) (which also falls under core pose), and STCS (Tang et al., 2024) (spatio-temporal clustering for socials). These are crucial for social interaction studies or high-throughput settings with group-housed animals. (3) 3D Pose-estimation: These go beyond 2D to reconstruct animal poses in three dimensios, often for more complex motor or biomechanical studies such as Anipose (Karashchuk et al., 2021), CAPTURE (Marshall et al., 2021), DANNCE (Dunn et al., 2021), and MouseVenus3D (Han et al., 2022). (4) Real-Time Tracking: Marker-less pose-estimation detection for closed-loop experiments, delivers posture-dependent stimuli by estimating animal pose online with millisecond latency, DeepLabStream (Schweihoff et al., 2021) is one of the tool for that purpose. (5) Behavior Classification: Tools that integrate a layer of identifying specific behaviors or actions from the pose data. Examples: SimBA (Nilsson et al., 2020) (which uses pose features to classify behaviors), BehaviorDEPOT (Gabriel et al., 2022), and ARBEL (Barkai et al., 2024). (6) Behavior Prediction: the highly specialized tools which forecast, flag abnormal behaviors [ABNet (Chen et al., 2024)] or detect pain [ARBEL (Barkai et al., 2024)] using trained models and pose dynamics. Even the prediction of future locomotion trajectories from past movements like FABEL (Catto et al., 2024). (7) Infrastructure/Frameworks: Foundation models for pose-estimation across species such as SuperAnimal (Ye et al., 2024).

The resulting distribution shows that core pose-estimation, multi-animal tracking, 3D pose-estimation, real-time tracking, behavior classification, behavior prediction, and infrastructure/frameworks are very prominent needs that many tools address. Overall, this analysis highlights that the tools are being developed with different end goals in mind.

### 4.3 Architectural approaches in tools

We evaluated the algorithmic and architectural approaches employed by the various pose-estimation tools (Table 4). Virtually all modern rodent pose-estimation tools leverage deep learning, but there are variations in network architecture and training strategies: A majority use Convolutional Neural Network (CNN) backbones originally developed for image recognition or human pose-estimation (Grinciunaite et al., 2016). For example, ResNet-50 is a common backbone (used in DLC and others), often coupled with deconvolution or upsampling layers to produce heatmaps for keypoint locations (Mathis et al., 2018). Some tools experimented with different backbones: AlphaTracker mentions DarkNet-53 and ResNet variants (Chen et al., 2023); LEAP used a variant of a stacked dense network (Pereira et al., 2019); similarly DeepPoseKit also uses variant of a stacked dense network and stacked hourglass model (Graving et al., 2019); newer tools like STPoseNet may integrate spatial transformer networks (Lv et al., 2024). For multi-animal tracking, architectures often incorporate an identity association component. SLEAP (Pereira et al., 2022), for instance, can use part affinity fields [similar to OpenPose (Cao et al., 2019)] or other graphical models to separate individuals. AlphaTracker’s pipeline combined a YOLO-based detection with pose-estimation, effectively splitting the task into detecting each animal and then finding key points (Chen et al., 2023).

**TABLE 4:** The tools are categorized based on the architecture family.

Architecture family	Tools
DeepLabCut-based (ResNet-50)	ABNet (Chen et al., 2024) ARBEL (Barkai et al., 2024) BehaviorDEPOT (Gabriel et al., 2022) DeepLabCut (Mathis et al., 2018) DeepLabStream (Schweihoff et al., 2021) DeepOF (Bordes et al., 2023) Keypoint-MoSeq (Weinreb et al., 2024) REVEALS (Phadke et al., 2024) SaLSa (Sakata, 2023) SimBa (Nilsson et al., 2020)
YOLO-based	ContrastivePose (Zhou et al., 2023) Seizure Classification Pipeline (Yu et al., 2025) STCS (Tang et al., 2024)
YOLOv3-based	AlphaTracker (Chen et al., 2023)
YOLOv8-based	STPoseNet (Lv et al., 2024)
SLEAP-based (UNet/Hourglass)	FABEL (Catto et al., 2024) SLEAP (Pereira et al., 2022)
Semi-supervised Bayesian Ensemble	Lightning Pose (Biderman et al., 2024)
Pretrained ensemble (DeepLabCut Model)	SuperAnimal (Ye et al., 2024)
**Other CNN**	
Multi-view CNN	MouseVenue3D (Han et al., 2022)
Custom 15-layer CNN	LEAP (Pereira et al., 2019)
3D triangulation TCN	CAPTURE (Marshall et al., 2021)
CNN (FlexibleBaseline) Optical Flow	OptiFlex (Liu et al., 2021)
MSC-Multibox detector 8-stack Hourglass CNN	MARS (Segalin et al., 2021)
Custom 3D pose CNN	Temporal Semi-supervision Method (Li et al., 2023)

### 4.4 Accuracy versus speed trade-offs

One practical consideration in pose-estimation tool performance is the trade-off between accuracy and speed. We aggregated the performance information reported in tool papers to qualitatively assess this trade-off. Different studies report performance in different ways, but two common measures for pose-estimation accuracy [often quantified by metrics like% of correct key points, mean pixel error, or mAP (mean average precision)] and runtime efficiency [measured in FPS (frames per second) processed, or whether the method can run in real-time]. We summarize schematically how tools tend to position themselves, available in Supplementary Tables 1–3. Generally, most tools cluster toward the high-accuracy end, given the emphasis on precise tracking in research. In the absence of evaluation against a standardized benchmark dataset, the reported performance values in their studies are not directly comparable and should be interpreted as arbitrary. However, a subset extends toward the all-rounders as most of the tools are built upon DLC-based architecture.

### 4.5 Chronological list of methods

**FIGURE 3:**
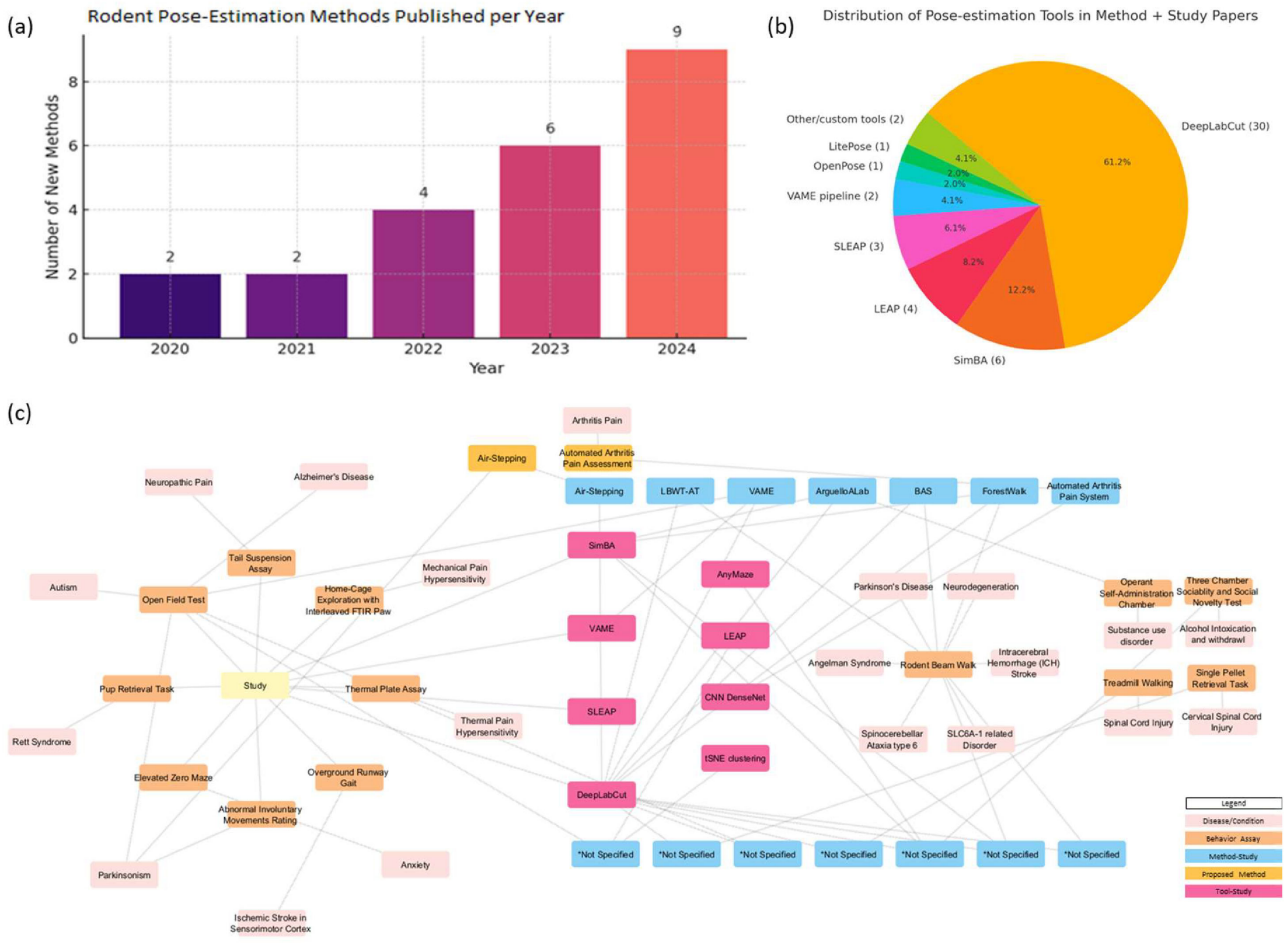
**(a)** Chronological milestone for method-focused studies. **(b)** Marker-less pose-estimation tool usage frequency across method development and disease studies. **(c)** The cross-analysis map of disease conditions, behavioral assays, study-focused or method-focused studies, and tools to present the current scenario of marker-less pose-estimation technology in the disease studies.

We also looked at the timeline of the Method-focused studies to see when researchers started incorporating pose-estimation into their experimental methods (Figure 3a). Unlike the tool development, which began in the 2018, the uptake of pose-estimation in general behavioral research shows a slight delay. The earliest Method category papers in our review were published around 2020. There was only one such study in 2020 that met our criteria and a couple in 2021. The count rises in 2022 and more sharply in 2023 and 2024, similar to the tool trend. Many published methods were focused on diverse behavioral assays such as gait analysis (Li et al., 2022; Sheppard et al., 2022), maternal care (Lapp et al., 2023), social interaction (Sterley et al., 2024; Zahran et al., 2024), and pain behavior (Li et al., 2024). Notably, 2024 emerged as a peak year, with a considerable number of new pipelines introduced. And the momentum appears to continue into 2025 (we already have five from early 2025 in our inclusion list). This timeline suggests that broad adoption by experimentalists lagged a year or two behind the introduction of major tools. It makes sense: tools like DLC (Mathis et al., 2018) became widely known around 2018–2019, and after some time for dissemination and training, more labs began applying them to their own experiments, leading to publications a year or more later. The accelerating trend in 2023–2024 indicates that pose-estimation is becoming more mainstream in the methods of behavioral labs. This analysis highlights the encouraging fact that the community is increasingly embracing these new methods, though it also points to a gap, it took a few years for many researchers to integrate these tools, suggesting a learning curve or initial resource barrier that needed to be overcome.

### 4.6 Pose-estimation tools used in method papers

We systematically reviewed the tools used for pose-estimation in both Method and Study category papers (37 papers total). As discussed earlier for method studies, DLC was the most commonly used tool (Figure 3b). DLC was used in 30 of 37 studies (∼81%). This includes various versions (2D, 3D, multi-animal extensions) but collectively underscores its prevalence. Most of the method papers explicitly used DLC (Mathis et al., 2018) for their pose tracking, often in conjunction with downstream analysis frameworks such as SimBA (Nilsson et al., 2020). For example, tools like AMBER (Lapp et al., 2023), ArguelloALab (Sanabria et al., 2025), and BAS (Piotrowski et al., 2024) all incorporated DLC keypoints and used Random Forest classifiers for behavior annotation. Several tools also employed hybrid approaches by integrating pose-estimation with domain-specific algorithms. For instance, Air-Stepping employed circular statistics and EMG step-matching (Mistretta et al., 2024), while Posture Analysis Workflow relied on FluoRender scripts for beam-walk kinematics (Wan et al., 2023). While performance reporting varied in detail, pixel error was the most common accuracy metric. Tools such as ForestWalk (Tozzi et al., 2025) and ArguelloALab (Sanabria et al., 2025) reported test errors ranging between 3–10 pixels, demonstrating practical precision for behavioral quantification in freely moving animals. DLC was the prominent framework found in the developments of the above-mentioned methods. Bias toward DLC may arise from its position as the earliest developed tool in the field development and established role as a benchmark tool in the field. And based on our findings, most of the behavior classification pipeline, and methodology are built upon DLC, using it as the foundational keypoint detection framework, followed by the application of specialized algorithms to address specific research problems. From a broader perspective, this indicates that researchers doing rodent experiments largely rely on a few well-established pose-estimation platforms rather than exploring the trend or writing their own from scratch.

LEAP (Pereira et al., 2019) was used in about four studies (mostly older ones before 2020) and SLEAP (Pereira et al., 2022) appearing in a few studies (approximately 3 out of 37). Those were typical cases needing multi-animal tracking or where authors were early adopters of this newer tool. No other single tool besides DLC and SLEAP showed up more than once or twice in the method and studies. This indicates that the community has largely coalesced around one primary tool (DLC) for conducting pose-estimation in practice; the usage frequency we see reflects a lag: tools introduced a few years ago (like DLC, SLEAP) have usage now, whereas brand-new ones have little to no representation yet outside their own introduction papers. This metric also highlights a potential risk: with so much reliance on one tool, if that tool had any biases or limitations, many results could be affected similarly. Over time, we may see diversification as newer tools mature and demonstrate clear advantages. As of the data in our review, the pose-estimation landscape in practice is highly centered on DLC.

### 4.7 Behavioral assays addressed by method papers

We analyzed which behavioral tests or paradigms were most commonly addressed by the Method-focused studies (Table 5). This gives insight into where researchers find pose-estimation most useful in terms of types of behavior. The rodent balance beam walk (and similar gait/coordination tests) emerged as a frequently used paradigm in these papers. At least four independent method studies focused on beam walking tasks to evaluate motor coordination, often in the context of neurological disorders (Lang et al., 2020; Tozzi et al., 2025) or injuries (Ruiz-Vitte et al., 2025). Pose-estimation is particularly well-suited here because it can count foot slips, measure crossing speed, and even detail how each paw moves – critical for detecting ataxia (Lang et al., 2020) or subtle motor deficits (Tozzi et al., 2025). Open field testing was another common assay, appearing either alone or in combination with other tests in several studies. In an open field, pose tracking gives not only total distance and speed (which could be done with simpler tracking) but also posture, limb movement patterns, and specific behaviors like rearing if 3D or multi-point is tracked (Klibaite et al., 2022). Some studies used open-field data to derive more complex metrics (such as unsupervised clustering of movement motifs) (Klibaite et al., 2022; Bogachev et al., 2023; Miller et al., 2024). Elevated Plus Maze and related anxiety tests (Sharma et al., 2025) (light-dark box, etc.) were present in a few papers. Pose-estimation here can automate measurements such as time spent in open vs. closed arms, as well as provide additional detail like head dips or stretch-attend postures if key points are tracked. Forced Swim Test (FST) was included in at least one study (Zhai et al., 2025). Social interaction tests and Operant behavior tests were featured in a couple of method papers. At least one method paper specifically dealt with an arthritis pain model (Li et al., 2024), using pose tracking to evaluate gait changes and pain-related behaviors like weight shifting. Another included a pain test in a broader context (Norris et al., 2023). A minority of studies looked at naturalistic behaviors (like maternal care or freely moving in homecage behaviors) for continuous pose tracking to capture subtle or long-term patterns (Lapp et al., 2023). This suggests that the community finds immediate value in applying pose tracking to tasks where movement is central, and deficits are quantitative. Other domains (social and cognitive tests) are less represented, possibly because they are either harder to quantify or just emerging areas for such analysis. In time, as pose-estimation becomes more routine, we might see it applied even more broadly.

**TABLE 5:** Developed methods primarily focusing on disease conditions.

Behavioral domain	Behavioral assay	Method papers	Associated disease models
Locomotion and gait	Open Field Test	8	Alzheimer’s disease (Bogachev et al., 2023; Miller et al., 2024)
	Rodent Beam Walk	5	Autism (Klibaite et al., 2022)
	Treadmill/Runway Walking	2	Angelman syndrome (Tozzi et al., 2025)
	Air-Stepping (Neonatal)	1	Spinocerebellar/Ataxia (Lang et al., 2020; Piotrowski et al., 2024)
	KineWheel Gait Task	1	Stroke (Weber et al., 2022; Ruiz-Vitte et al., 2025)
	Elevated Plus Maze	2	Parkinson’s disease (Mistretta et al., 2024)
			Spinal cord Injury (O’Neill et al., 2022; Sato et al., 2022)
			SLC6A-1 disorder (Tozzi et al., 2025)
Anxiety-like behavior	Elevated Zero Maze	1	Anxiety-like Behavior (Sharma et al., 2025)
Rewards and operant conditioning	Operant/Reward Tasks	1	Cocaine self-administration (Sanabria et al., 2025)
	Three Chamber Sociability and Social Novelty Test	1	Alcohol consumption preference (Zahran et al., 2024)
Maternal care	Maternal Behavior	1	Maternal care deficits (Lapp et al., 2023)
Pain assessment	Arthritis Pain Gait Assay	1	Arthritis (Li et al., 2024)
Fine motor skills	Single-Pellet Reaching	1	Stroke Recovery (Weber et al., 2022)
	String-Pulling	1	Motor Deficit Models (Sandhu et al., 2024)
Spontaneous/longitudinal behavior	Homecage Observations	1	Stroke Recovery (Weber et al., 2022)

### 4.8 Disease models addressed by method papers

Among the Method-focused studies, a significant subset involved specific disease or injury models. We tallied the types of disease models featured (Table 5). Out of the 28 method papers, 14 (50%) incorporated an explicit disease or physiological challenge. The distribution of disease models in the method papers included: (1) Neurodegenerative and Neurological Disorders: Several studies focused on models of diseases such as Parkinson’s disease (Yang et al., 2024), Alzheimer’s disease (Bogachev et al., 2023; Miller et al., 2024), and spinocerebellar ataxia (Piotrowski et al., 2024). (2) Neurodevelopmental Disorders: Autism spectrum disorder models (Klibaite et al., 2022) and other developmental disorder models like Angelman syndrome (Tozzi et al., 2025) appeared in method studies. (3) Psychiatric/Addiction Models: Some papers involved substance use or withdrawal models [such as chronic alcohol exposure and withdrawal in mice (Zahran et al., 2024) and cocaine self-administration in rats (Sanabria et al., 2025)]. These studies used pose-estimation to observe changes in behavior, such as locomotor activity or specific actions during withdrawal periods. (4) Pain and Injury Models: Chronic pain models [like inflammatory arthritis in mice (Li et al., 2024)] and acute injury models [like spinal cord injury (Sato et al., 2022) or stroke (Weber et al., 2022)] were present. The stroke model study, for instance, introduced a new method to assess motor recovery by tracking limb movement symmetry in a home cage monitoring (HCM) (Ruiz-Vitte et al., 2025), the rationale focuses on the idea of automated behavior analysis. From data presented it is evident that neurological disease models form the largest group among these method papers. This aligns with the intuition that motor deficits are a key feature of many neurological disorders, making those models a prime target for such methods.

### 4.9 Mapping behaviors to disease

The network is illustrated in Figure 3c represent the map linking behavioral assays to the disease models they were used to evaluate, across all relevant studies (both Method and Study categories). This mapping helps reveal if certain behaviors are particularly associated with certain types of disease research when using pose-estimation. We found that motor coordination tests like the beam walk are commonly used as mentioned above. These diseases naturally affect coordination, so researchers often employ beam walking or similar gait tests to quantify deficits (Bidgood et al., 2024; Ruiz-Vitte et al., 2025; Tozzi et al., 2025). Open field tests were used in a broad range of contexts, including Alzheimer’s models, autism models, and as baseline in many others. Social interaction tests were specifically utilized in neurodevelopmental disorder studies and occasionally in neurodegeneration or psychiatric models. Tests commonly used in the domain of Anxiety research like elevated plus maze appeared in contexts like Alzheimer’s disease (Miller et al., 2024) and in substance withdrawal studies (Sharma et al., 2025).

Pain-related gait assays (like automated scoring of limp or weight distribution) were obviously tied to pain models or nerve injury models (Norris et al., 2023). Complex behavior batteries were often used for models with uncertain phenotypes. For example, one comprehensive study on a Rett syndrome model mouse used a battery including open field, social test, and motor tests to capture a spectrum of behaviors via pose tracking (Mykins et al., 2024). The mapping highlights that each disease domain tends to utilize a relevant subset of behavioral tests, and pose-estimation is flexible enough to be applied to all these.

### 4.10 Limitation of screening strategy

The screening strategy leans extensively on automated processes from keyword searches to the retrieval of relevant publications. Publish or Perish software efficiently retrieves references, but it has limitations including a limit on results per query. Moreover, some retrieved entries lack abstracts, they might get prematurely excluded in subsequent screening step. ASReview relies on the structured abstracts and limits the inclusion of potentially relevant studies with missing or malformed metadata. Automated screening fell short when applied to extensive datasets. Notably, key studies such as Anipose (Karashchuk et al., 2021), A-SOiD (Tillmann et al., 2024), DANNCE (Dunn et al., 2021), and DeepPoseKit (Graving et al., 2019) were missed during the automated retrieval process.

## 5 Discussion

Our systematic review illustrates a period of rapid development and refinement of pose-estimation tools for rodent research. Over roughly 8 years (2017–2025), the field progressed from a handful of pioneering methods to a suite of sophisticated algorithms with diverse capabilities. Early tools established the feasibility of accurate marker-less tracking for instance, achieving sub-centimeter accuracy in detecting rodent limb positions effectively proving that computer vision could automate what was once manual scoring or marker-based motion capture (Mathis et al., 2018). Building on that foundation, successive tools have incrementally expanded the frontiers: introducing multi-animal tracking (Lauer et al., 2022) (to handle social groups or littermates in a cage), improving tracking speed (Phadke et al., 2024) (to approach real-time feedback, important for closed-loop experiments), and incorporating behavior classification and unsupervised analysis (Nilsson et al., 2020) (to interpret the raw pose data in terms of meaningful actions or patterns). The architectural evolution of these tools has been largely driven by advances in deep learning. Convolutional neural networks pre-trained on large datasets brought a step-change in keypoint detection accuracy around 2018, and since then, many tools have repurposed. The development trajectory of pose-estimation tools, from general frameworks like DLC (Mathis et al., 2018) and SLEAP (Pereira et al., 2022) to more specialized ones has been especially productive, developing a range of options that collectively cover many needs of the research community. The uptake of pose-estimation technology in preclinical behavioral experiments is clearly underway, as evidenced by the growing number of studies incorporating these methods. Our results show that starting around 2020, researchers began to apply pose tracking in classical rodent behavioral tests, and the trend has accelerated in the last few years. For example, in a open field test, instead of just recording the total distance travelled, researchers can quantify detailed trajectories, speed profiles, and even specific behavior postures automatically (Weber et al., 2022). In coordination tasks like the balance beam (Tozzi et al., 2025), subtle differences in how a rodent places its paws or maintains balance, which might be overlooked by human observation, are captured through automated video analysis (Ruiz-Vitte et al., 2025). This not only increases the sensitivity of the experiments (allowing detection of mild phenotypic differences or drug effects) but also their reliability [reducing observer bias and variability (Ruiz-Vitte et al., 2025)].

Our review highlights the use of pose-estimation to augment traditional behavioral assays rather than replace them outright. We see a methodological modernization in rodent behavioral science: the field is moving from stopwatches and manual counts toward automated, quantitative behavioral phenotyping. One of the most compelling findings of this review is how pose-estimation is empowering research on rodent models of disease. A transgenic mouse modeling early-stage Parkinson’s disease might not exhibit obvious motor deficits under casual observation, but with pose-estimation, researchers can detect slight irregularities in gait or balance that herald disease onset (Mistretta et al., 2024). Such sensitive measures can serve as early biomarkers of disease progression or as endpoints to test intervention efficacy that would otherwise require a much larger sample sizes and sophisticated experimentation to even notice. In pain and injury models, pose-estimation has allowed more objective and continuous monitoring of pain-related behaviors (Li et al., 2024). For instance, instead of relying solely on discrete scoring (like a pain scale based on observations at intervals), some studies continuously track how an animal shifts weight or adjusts posture, which can quantify pain levels with higher resolution over time (Norris et al., 2023). The ability to link specific behavioral metrics to disease conditions has much broader implications.

## 6 Limitation and future direction

While our review underscores many positive developments, it is important to acknowledge its limitations, as well as those in the field, and to suggest areas for future work. First, our review is limited by the scope of available literature, our search strategy, and the AI-assisted screening method. Being overly dependent on tools contains blind spots, which leads to the exclusion relevant studies as mentioned in the section above. And we focused on publications up to early 2016 and only those in English. It is possible that some relevant studies (especially very recent or non-indexed ones or those in other languages) were not captured. Second, the heterogeneity in study designs due to the lack of benchmarks across the included papers. This made it challenging to directly compare outcomes like accuracy or effect sizes. We relied on authors’ reported metrics; some provided comprehensive evaluations, while others offered only qualitative assessments. A more standardized benchmarking across tools would greatly facilitate objective comparison. In our synthesis, we had to qualitatively assess trade-offs and trends rather than perform a quantitative meta-analysis due to this variability. Third, our categorization into tool-focused, method-focused, and study-focused studies was somewhat subjective and there is overlap between categories. Some tool papers also performed biological experiments to showcase their tool; some method papers introduced minor technical innovations. We chose categories to structure the review. This also reflects a limitation in the field: interdisciplinary studies sometimes defy neat categorization and valuable insights might be get over looked.

In terms of the research field’s limitations revealed by this review, one notable aspect is the uneven adoption of pose-estimation. A large proportion of studies still come from either technology-oriented groups or early adopters instead of the preclinical research labs. Many traditional behavioral studies have yet to incorporate these methods. Barriers might include required expertise, computational resources, or simply inertia with established methods, as mentioned above. Another limitation is that while pose-estimation greatly improves measurement, it doesn’t automatically interpret behavior; behavioral meaning must be inferred from pose data, and that still relies on expert knowledge or complementary experiments. Advanced analytics like machine learning classification or unsupervised clustering can help identify patterns, but there’s a risk of over-reliance on algorithms without biological context. Future research should consider on linking pose-derived metrics more tightly to benchmark processes.

Looking ahead, future directions could include: (1) Expanding pose-estimation to more complex environments, most current studies are in relatively controlled settings. Adaptive methods are required (Pereira et al., 2022). (2) Enhancing 3D pose-estimation for rodents; a few studies did this, but it’s not widespread; improved 3D tracking could yield better readouts of complex behaviors (Lauer et al., 2022). (3) Integration with other data modalities such combining pose data with neural recordings, optogenetics triggers, or physiological readouts could provide a better understanding of behavior in context, and synchronized channel serve as a critical proof of concept, which are essential in establishing the benchmarks.

The advancement of pose-estimation has catalyzed progress in connected technologies, notably home cage monitoring (HCM) systems, which allow for 24/7, non-invasive collection of behavioral and physiological digital biomarkers (Baran et al., 2022). More recently, large language and vision-language models such as MouseGPT (Xu et al., 2025) and AmadeusGPT (Ye et al., 2023) have transformed behavior classification by directly interpreting raw video into open-vocabulary behavioral annotations also not relying on keypoint detection, steering the preclinical research toward new heights.

The current review’s limitations are natural product of a new and very fast-moving field. We attempted to compile a comprehensive overview, and while some gaps remain, the trends identified are well defined. A continued push to upgrade these tools and broaden their adoption will help ensure that the insights from pose-estimation reach their full potential in advancing behavioral science.

## 7 Conclusion

Marker-less pose-estimation is the advent of advanced pose-estimation techniques, though this transformation is still in progress. We found that the development of new pose-estimation tools has been vigorous over the last several years, providing researchers with unprecedented capabilities to track and quantify behavior. The adoption of these tools in experimental studies is growing, particularly in areas where fine behavioral details matter, such as disease models and complex behavioral assays. Despite the availability of these tools, our review also highlights a persisting gap between technological advancements and their implement. Many rodent studies have yet to incorporate marker-less pose-estimation. And the majority of practitioners rely on a few key software tools. The evidence from the studies we synthesized indicates that embracing marker-less pose-estimation can significantly enhance the quality of data and conclusions in preclinical research. Whether it is validating a new therapy in a mouse model of disease or exploring fundamental questions of neuroscience, the ability to quantify behavior with high resolution leads to more robust and reproducible findings. In summary, the current trend in pose-estimation for rodent models is one of promising growth. Continued advancement is mostly likely to be the case. However, collaboration between tool developers and traditional researchers, for addressing practical barriers will be essential. By doing so, the field can ensure that the considerable advances in computational behavior analysis fully translate into deeper insights and breakthroughs in biomedical research.

## Data Availability

The raw data supporting the conclusions of this article will be made available by the authors, without undue reservation.
