# Supplementary material for: Does advancement in marker-less pose-estimation mean more quality research? A systematic review
Source: Front Behav Neurosci. 2025 Aug 22;19:1663089. doi: 10.3389/fnbeh.2025.1663089 (PMC12411509; doi:10.3389/fnbeh.2025.1663089)
Supplement: Supplementary file 1 [file Data_Sheet_1.docx]

Supplementary Figure:

Figure 1:


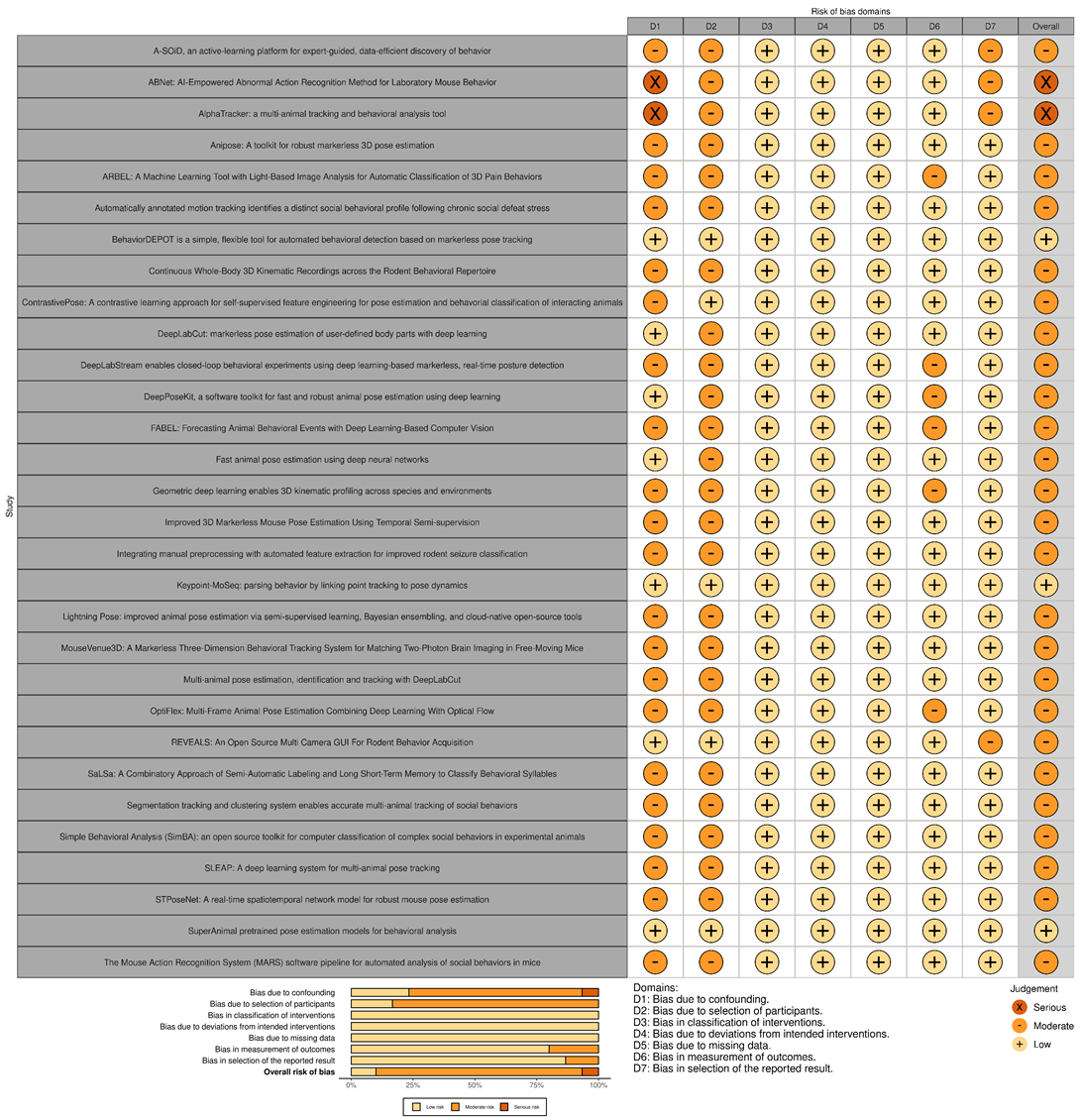


**Figure 1.** Risk of bias analysis: plot and summary of the selected studies categorized as Tool-focused (n = 30).

Figure 2:


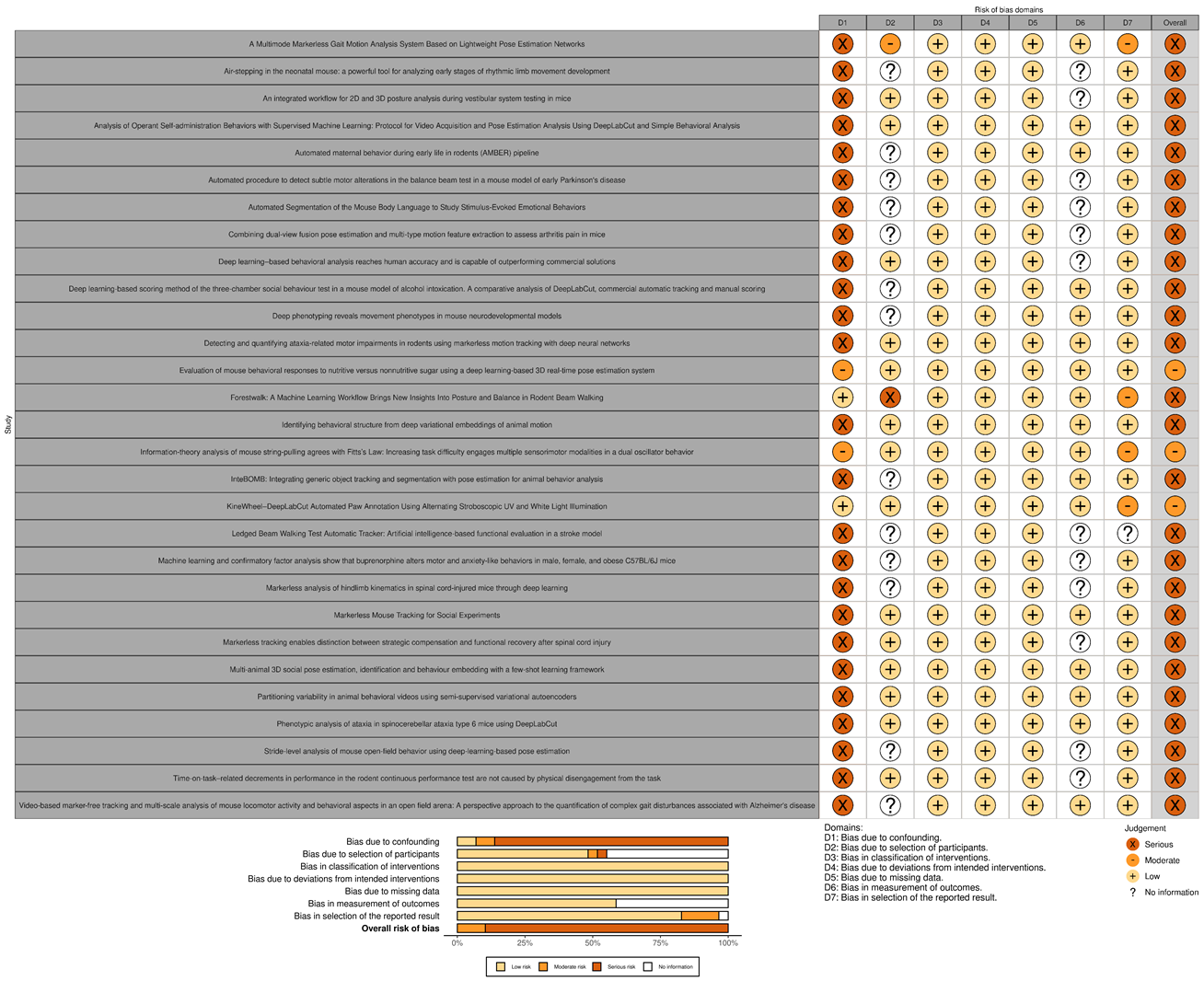


**Figure 2.** Risk of bias analysis: plot and summary of the selected studies categorized as Method-focused (n=28)

Figure 3:


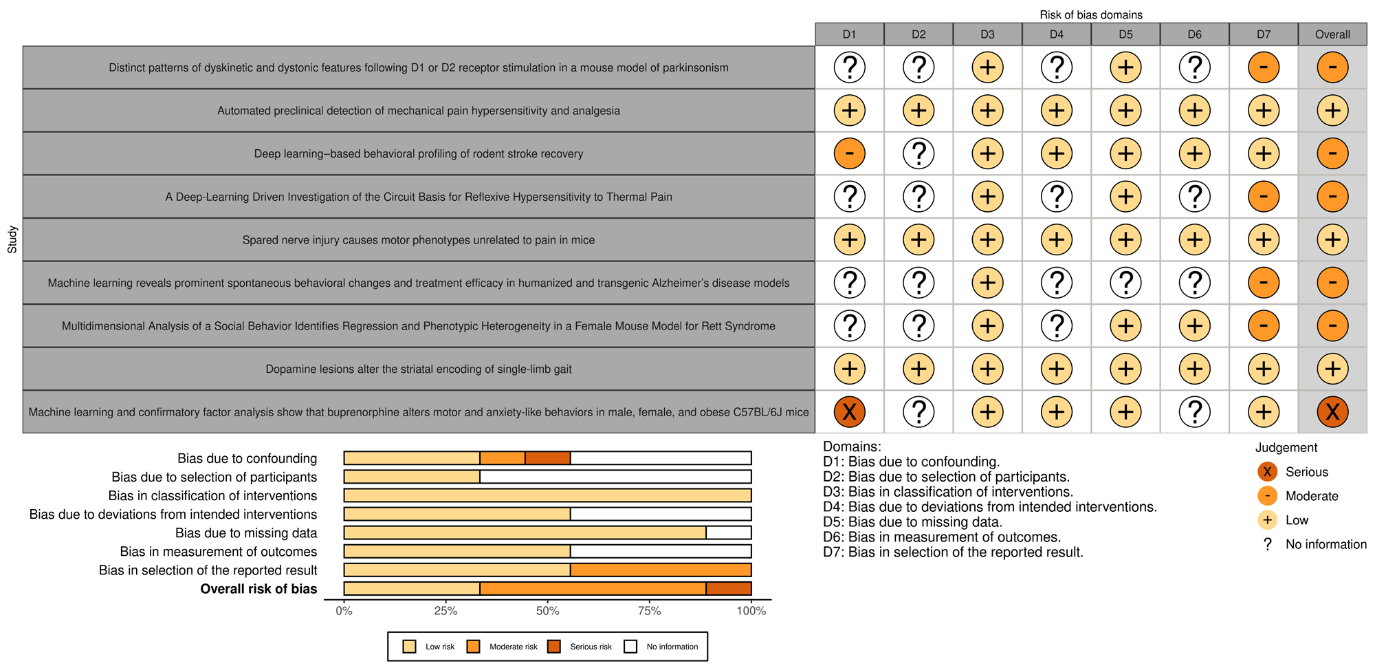


**Figure 3.** Risk of bias analysis: plot and summary of the selected studies categorized as Studies-focused (n=9)
